# Ubiquitous *Gasp1* overexpression in mice leads mainly to a hypermuscular phenotype

**DOI:** 10.1186/1471-2164-13-541

**Published:** 2012-10-10

**Authors:** Olivier Monestier, Caroline Brun, Katy Heu, Bruno Passet, Mélanie Malhouroux, Laetitia Magnol, Jean-Luc Vilotte, Véronique Blanquet

**Affiliations:** 1 INRA, UMR1061 Unité de Génétique Moléculaire Animale, Limoges, 87060, France; 2 Université de Limoges, Limoges, 87060, France; 3 INRA, UMR1313 GABI Génétique Animale et Biologie Intégrative, Jouy-en-Josas, 78352, France

## Abstract

**Background:**

Myostatin, a member of the TGFβ superfamily, is well known as a potent and specific negative regulator of muscle growth. Targeting the myostatin signalling pathway may offer promising therapeutic strategies for the treatment of muscle-wasting disorders. In the last decade, various myostatin-binding proteins have been identified to be able to inhibit myostatin activity. One of these is GASP1 (Growth and Differentiation Factor-Associated Serum Protein-1), a protein containing a follistatin domain as well as multiple domains associated with protease inhibitors. Despite *in vitro* data, remarkably little is known about *in vivo* functions of *Gasp1*. To further address the role of GASP1 during mouse development and in adulthood, we generated a gain-of-function transgenic mouse model that overexpresses *Gasp1* under transcriptional control of the human cytomegalovirus immediate-early promoter/enhancer.

**Results:**

Overexpression of *Gasp1* led to an increase in muscle mass observed not before day 15 of postnatal life. The surGasp1 transgenic mice did not display any other gross abnormality. Histological and morphometric analysis of surGasp1 *rectus femoris* muscles revealed an increase in myofiber size without a corresponding increase in myofiber number. Fiber-type distribution was unaltered. Interestingly, we do not detect a change in total fat mass and lean mass. These results differ from those for myostatin knockout mice, transgenic mice overexpressing the myostatin propeptide or follistatin which exhibit both muscle hypertrophy and hyperplasia, and show minimal fat deposition.

**Conclusions:**

Altogether, our data give new insight into the *in vivo* functions of *Gasp1*. As an extracellular regulatory factor in the myostatin signalling pathway, additional studies on GASP1 and its homolog GASP2 are required to elucidate the crosstalk between the different intrinsic inhibitors of the myostatin.

## Background

Improving muscle mass and function is of a considerable clinical interest in therapeutic strategies for musculoskeletal disorders and has been assessed by several studies [1-3]. In the last decade, among all these approaches, dramatic attention has been focused on the regulation of the myostatin (GDF8) pathway. Indeed, myostatin is a key regulator of skeletal muscle growth and homeostasis. In mice, targeted inactivation of the *Gdf8* gene causes a large and widespread increase in skeletal muscle mass, resulting from a combination of muscle cell hyperplasia and hypertrophy. Moreover, postnatal inhibition of myostatin signalling, through the delivery of neutralizing antibodies, myostatin propeptide injection or antisense RNA showed skeletal muscle improvement when administered to mice of different ages [4-12]. The identification of myostatin-binding proteins capable of regulating myostatin activity further expanded the number of potential therapeutic targets [13]. Thus, follistatin (FS) can function as a potent myostatin antagonist, its overexpression in mice is found to enhance muscle growth [13,14]. The increase in muscle mass observed in transgenic mice overexpressing FS in muscle is even significantly larger than that observed in *Gdf8*^*-/-*^ mice [15]. However, follistatin is not a specific inhibitor for myostatin and binds also to other TGFβ including activin. In addition to follistatin, two other proteins have been identified that are involved in the regulation of the myostatin. Follistatin-related gene is highly similar to follistatin and has also been shown to inhibit activin and multiple bone morphogenic proteins *in vitro*[16]. Growth and differentiation factor-associated serum protein-1 (GASP1; also called WFIKKN2) is a secreted protein that contains multiple domains associated with protease-inhibitory proteins including a whey acidic protein domain, a Kazal domain, two Kunitz domains, and a netrin domain. It also contains a highly conserved module of cysteine-rich sequence termed the follistatin domain. GASP1 was shown to bind directly but independently to both mature myostatin and the myostatin propeptide and to inhibit myostatin activity but not that of activin or TGFβ1 *in vitro*[17]. Like its homologous protein GASP2, GASP1 also has a high affinity for GDF11, a secreted factor that regulates anterior/posterior patterning in the axial skeleton [18]. Recent studies showed that both GASP1 and GASP2 bind growth factors TGFβ1, BMP2 and BMP4 but do not inhibit *in vitro* their signalling activity [19]. GASP1 and 2 show distinct expression patterns both in the developing fetus and the adult. In the developing fetus, GASP1 expression is highest in the brain, skeletal muscle, thymus and kidney while GASP2 is abundant in the lung, skeletal muscle and liver [20]. In the adult, GASP1 is primarily expressed in the ovary, testis, and brain while GASP2 is in the pancreas, liver, and thymus [20]. To date, despite these data, little is known about the precise *in vivo* functions and protein interactions of these GASP proteins. To highlight the range and extent of *Gasp1* roles during mouse development and in adulthood, we have generated and characterized transgenic mouse lines that ubiquitously overexpress *Gasp1* under the control of a cytomegalovirus (CMV) promoter. Six transgenic lines have been isolated and two were selected with different levels of overexpression of *Gasp1* in muscle, brain, heart, spleen, liver, lung and kidney for analyses. Detailed phenotypic characterization shows muscle abnormalities but no obvious defects in other major organ systems.

## Results

### Generation of *Gasp1* transgenic mice

**Figure 1:**
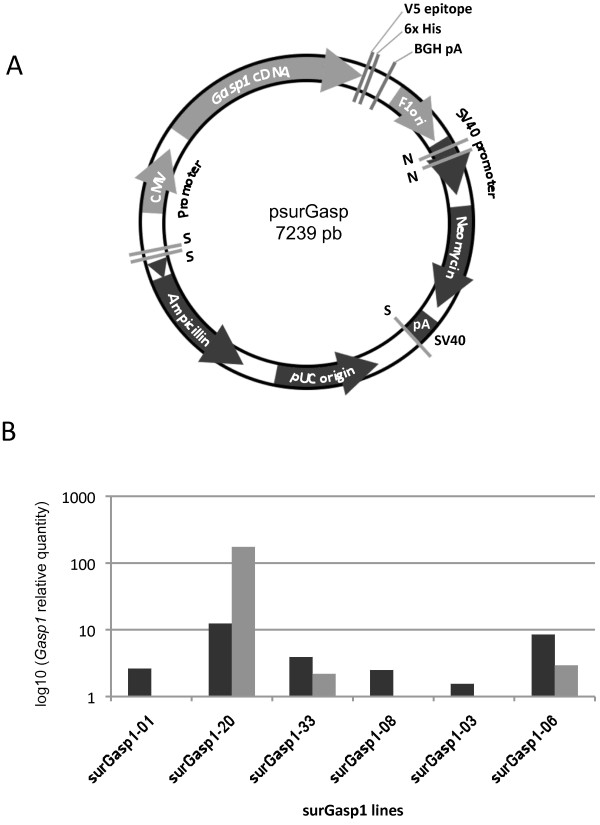
**Generation of the surGasp1 transgenic mice. A**) Schematic illustration of the surGasp1 transgene construct. To generate transgenic mice, a 3578 bp SalI-NsiI fragment comprising the *Gasp1* coding sequence under the CMV promoter was injected into pronucleus of the one cell mouse embryo. S:SalI ; N:NsiI. **B**) Quantitative PCR analysis of *Gasp1* expression on muscle (light grey) and brain (dark grey) samples from F1 mice of the six independent transgenic lines generated.

**Figure 2:**
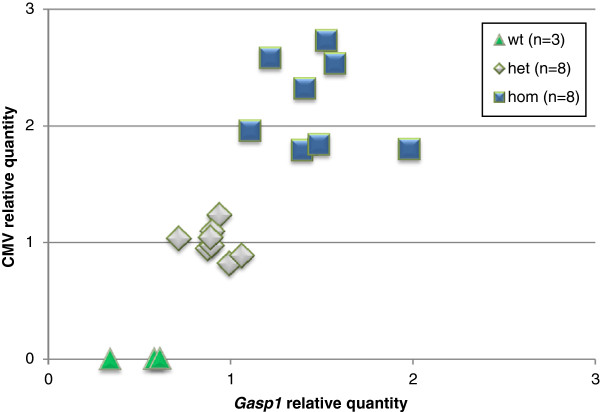
**Semi quantitative real-time PCR based genotyping.** The graph represents the genotyping of F2 surGasp1-20 or surGasp1-06 mice. The abscissa shows the relative quantification of *Gasp1*, the ordinate corresponds to the CMV relative quantification. Animals can be divided in 3 groups according to their CMV and *Gasp1* DNA copy number: wildtype animals [triangle, (n = 3)] are characterized by no amplification of the CMV sequence, heterozygous mice [rhombus, (n = 8)] have the same CMV or *Gasp1* copy number as the F1 animals used as calibrator, and homozygous mice [square, (n = 8)] have twice as many CMV and *Gasp1* copies as F1 animals.

With the purpose of developing transgenic mice with constitutive expression of *Gasp1*, the cDNA was cloned into a vector containing a CMV promoter. The transgene was liberated from the vector backbone by the enzymes SalI and NsiI as shown in Figure 1A and was purified for microinjection. After reimplantation of the manipulated embryos, pups were screened for the presence of the transgene by PCR analyses of genomic DNA from tail clips to identify those harboring the transgene DNA. Six of the 39 offspring carried the transgene construct in the genome. Breeding lines were successfully established from all 6 founders by crossing them with a non-transgenic mouse. These lines were designated surGasp1-01, surGasp1-03, surGasp1-06, surGasp1-08, surGasp1-20 and surGasp1-33. Analysis of the F1 offspring showed that the transgene was for each line transmitted to ~ 50% of the litter as expected for Mendelian inheritance. Quantitative polymerase chain reaction was performed to determine *Gasp1* mRNA levels from muscle and brain of F1 animals (Figure 1B). As predicted, expression levels varied depending of the transgenic strain. We opted to pursue further phenotypical analyses on the two lines surGasp1-20 and surGasp1-06 because of their high *Gasp1* expression levels in both tissues. For each line, heterozygous male and female mice were crossed to produce F2 mice. About 75% of the F2 offspring carried the transgene. To determine their precise genotype, heterozygote or homozygote, and to estimate the integrated transgene copy number, semi quantitative real time PCR was performed as shown in Figure 2. The homozygous surGasp1-20 mice harboured 4 copies of the *Gasp1* transgene while the surGasp1-06 mice had 8 copies. The copy number was stable within all subsequent generations.

### Tissue-specific expression

**Figure 3:**
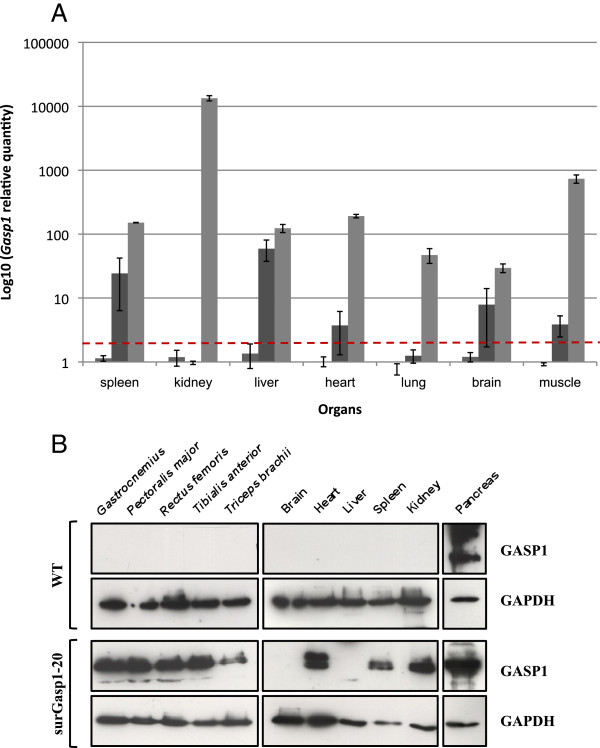
**Relative expression levels of *Gasp1 *RNAs in mouse tissues. A**) mRNA expression levels were determined by qRT–PCR relative to the reference genes *Gapdh*, *TfIID* in extracts from 7 different mouse tissues from wild-type (grey, n = 4), surGasp1-20 (light grey, n = 4), surGasp1-06 (dark grey, n = 4) animals. The horizontal dashed line represents a twofold increase in expression level. Mean values of three separate data acquisitions per tissue sample are shown. Data are expressed as mean +/- SEM. **B**) Western blot analysis of GASP1 expression in surGasp1 transgenic mice: total proteins were extracted from spleen, kidney, liver, heart, pancreas, brain and some skeletal muscles dissected from 12-week-old mice. GASP1 was detected using a polyclonal goat anti-GASP1. Secondary HRP-anti-goat IgG was used for signal visualization with chemiluminescence. GAPDH was used as a loading control. The two protein bands (bottom panel) probably represent different glycosylation states of GASP1

In order to determine the tissue distribution of the transgene expression and its level, total RNA was isolated from multiple tissues from the offspring of the surGasp1-20 and surGasp1-06 breeder lines and was subjected to quantitative RT-PCR amplification. As shown in Figure 3, the surGasp1-20 line had the highest expression (50 to 10000 fold compared to wild-type FVB/N) in all tested organs with a very high increase in kidney. Although surGasp1-06 transgenic mice carried more copies of the transgene, they displayed a much lower *Gasp1* RNA expression level with no significant overexpression in kidney and lung. Western blot analysis of GASP1 expression in various tissues was performed using three different antibodies. We detected its expression in all surGasp1-20 tested tissues and only in the pancreas of wild-type animals (Figure 3B). No positive signal was revealed in the other tissues of wild-type or surGasp1-06 mice, most probably because of the low level of expression of the protein or the potential lack of sensitivity of our western analysis.

### Transgenic mice are characterized by an increase in muscle mass

**Figure 4:**
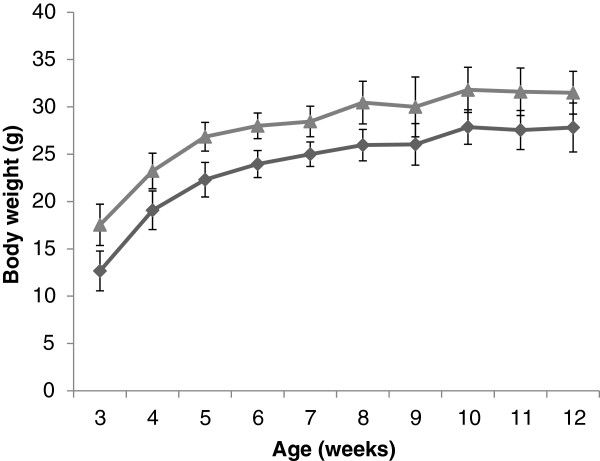
**Body weight of surGasp1 mice.** Body weight of wild type (dark grey, n = 40) and surGasp1-20 homozygous mice (light grey, n = 31) were measured from 3 to 12 weeks old. Data were expressed as mean +/- SD.

**Figure 5:**
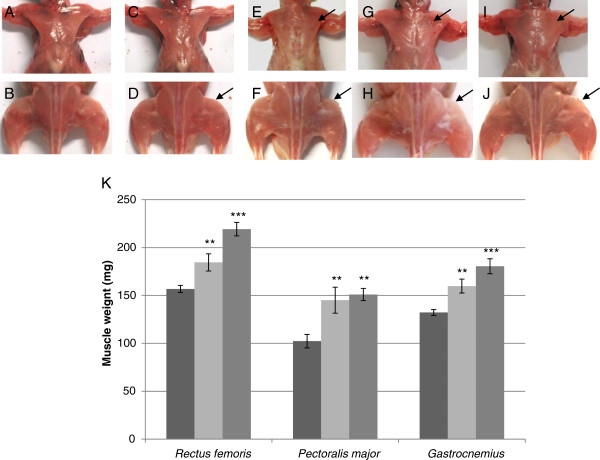
**Effects of *Gasp1 *overexpression in skeletal muscle.** Increased skeletal muscle mass in surGasp1-06 [heterozygote (**C**, **D**), homozygote (**E**, **F**)] mice or surGasp1-20 animals [heterozygote (**G**, **H**), homozygote (**I**, **J**)] compared to wildtype littermates (**A**, **B**). Top panels: pectoral muscles; bottom panels: lower limb. (**K**) Muscle weight. Muscles of 12-week-old mice were carefully dissected and weighed. Wild-type (dark grey) n = 36; heterozygotes surGasp1-20 (light grey) n = 27; homozygotes surGasp1-20 (grey) n = 18. Values are means ± SEM. * : p < 0.05; ** : p < 0.01; and *** : p < 0.001.

Immediately after birth, the transgenic mice appeared normal. However, within 2 weeks after birth, homozygotes surGasp1-20 mice showed a significant increase in body weight of about 4.5 grams compared to wildtype littermate mice (Figure 4). Such a difference was also detected in older mice (12-16 weeks old). A similar but milder phenotype was also observed in the surGasp1-06 line (data not shown). From 8 weeks old, mice overexpressing *Gasp1* showed a hypertrophic skeletal muscle phenotype (Figure 5A-J). Increased muscle mass was also evident when we compared the wet weights of the *gastrocnemius, rectus femoris* and *pectoralis major* muscle groups of heterozygous or homozygous surGasp1-20 mice with those of the wild-type mice (Figure 5K). Similar results were observed for surGasp1-06 muscles (data not shown). The increases were found in both sexes with a muscle weight gain of 35% for the *gastrocnemius* and *rectus femoris*, and 45% for the *pectoralis major* muscles from homozygous surGasp1-20 mice. The magnitude of these increases was correlated with the muscle expression level of the transgene. Surprisingly, no significant defect in the development of the other tested major organs (brain, heart, kidney, liver, spleen) was detected (data not shown).

### Effect of the overexpression of GASP1 on body composition

**Figure 6:**
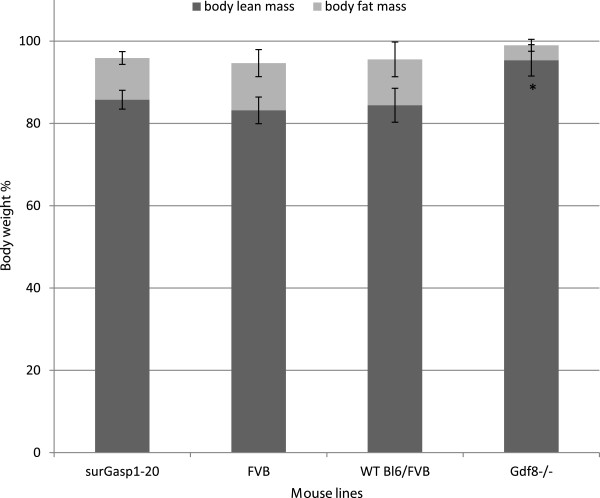
**Body composition of surGasp1 mice.** Proportion of body fat and lean mass in surGasp1-20, wild-type FVB or Bl6/FVB, myostastin deficient (*Gdf8*^-/-^) mice at 10 weeks was calculated as the percentage of their respective body weights. Since the *Gdf8*^-/-^ mice were maintained on the Bl6/FVB background, we used wild-type Bl6/FVB as control. Data are presented as means ± SEM. * : p < 0.05.

To determine the origin of increased body weight in surGasp1 mice, analysis of body composition by EchoMRI-500™ was performed allowing us to measure whole body fat mass, lean tissue mass, free water, and total body water in live animals without the need for anesthesia or sedation. This study revealed no change in total fat mass and lean mass in surGasp1-20 mice. It contrasts with data obtained with constitutive myostatin deficient mice which present a reduction in fat mass (Figure 6).

### The increase in muscle mass results from hypertrophy rather than hyperplasia

**Figure 7:**
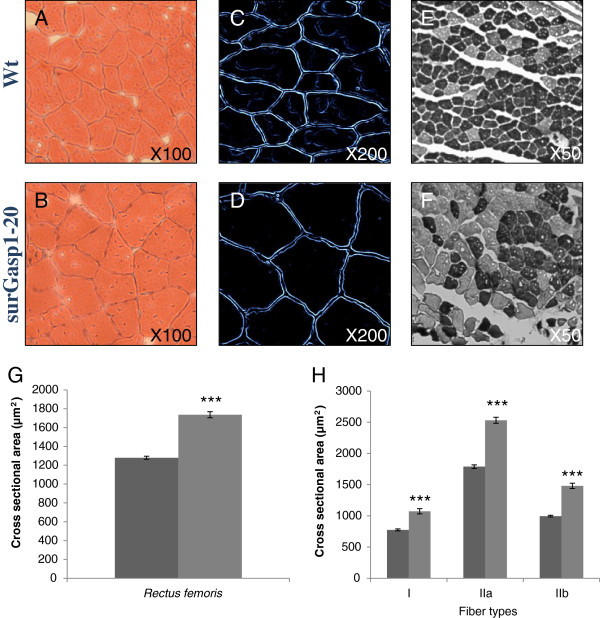
***Gasp1 *overexpression induces a marked muscle fiber hypertrophy.** Sections were prepared from *rectus femoris* muscles of wild-type or surGasp1-20 mice and stained by hematoxylin-eosin (**A**, **B**), silver impregnation (**C**, **D**) or myosin ATPase stain with acidic preincubation (**E**, **F**). (**G**) Overall muscle fiber cross-sectional area of wild-type or surGasp1-20 *rectus femoris* muscles. (**H**) Distribution of muscle fiber sizes in wild-type (dark grey) or surGasp-1-20 (light grey). *** Statistically significant compared to control, p < 0.001.

To determine whether the increase in muscle mass resulted from a combination of increased fiber numbers and increased fiber sizes, we performed histological and morphometric analysis of the *rectus femoris* muscles from wild-type (n = 3, 600 fibers per animal) and homozygotes surGasp1-20 mice (n = 3, 300 fibers per animal). Cryostat sections of muscles stained by hematoxylin-eosin or Gomori’s silver impregnation indicated a substantial increase (36% on average) in the fiber diameter of the transgenic mice (Figure 7A-D, G). Fiber typing was performed using the myosin ATPase method allowing differentiating 3 fiber types: I, IIA and IIB [21]. All muscle fiber types increased in diameter in the transgenic lines (Figure 7E, F, H and data not shown). The relative proportion of type I, II myofibers in wild-type, surGasp1-06 and surGasp1-20 *rectus femoris* muscle did not change significantly (Additional file 1). Furthermore, no difference was found in the total number of muscle fibers per cross-sections. These results suggested that increased muscle mass was due to hypertrophy rather than hyperplasia.

## Discussion

Intensive efforts have been made over the last decade to explore the molecular mechanisms underlying the regulation and function of myostatin, a key negative regulator of skeletal muscle growth. The identification of several components of the myostatin-signalling pathway had important implications with respect for testing the therapeutic value of a myostatin antagonist in muscle wasting disorders such as Duchenne muscular dystrophy [22,23] cachexia and age-related sarcopenia [24-26]. Among the known myostatin-binding proteins, GASP1 has been shown to inhibit myostatin activity *in vitro* and may maintain myostatin latency but no data was available on the effect of GASP1 when expressed as a transgene in all skeletal muscles of wild type mice. In the present study, we have generated a “gain of function” mouse model to further understand the *in vivo* roles of *Gasp1*. These mice carry a transgene containing the *Gasp1* coding sequence under the transcriptional regulation of the ubiquitous CMV promoter. As a consequence, *Gasp1* mRNA expression is greatly enhanced in several organs including muscle, brain, spleen, liver, heart, lung and kidney. Analysis of the two *Gasp1* transgenic lines with the highest transgene expression revealed a skeletal muscle hypertrophic phenotype. This might have been expected based on previous data indicating that GASP1 can function as a myostatin antagonist, similar to follistatin or the follistatin-related gene protein whose overexpression leads to an increase of the muscle mass. Furthermore, viral delivery of a *Gasp1* expression cassette into adult muscle has been shown to induce increases in muscle mass and grip strength [13]. The surGasp1 mice have similar average life span compared to standard inbred laboratory mice (http://research.jax.org/faculty/harrison/ger1vi_Lifespan.html) Except the phenotype described in this paper, no other gross abnormalities were noted, even in elder surGasp1 mice (≈ 28 months). Effects of elevated GASP1 on body growth were not observed before day 15 of postnatal life. The enhanced muscle growth occurs in both male and female animals with a more pronounced phenotype in male *pectoralis major* muscle. The individual weights of the *gastrocnemius*, *rectus femoris* and *pectoralis major* muscles were increased. The masses of other skeletal muscles were also increased. The sizes of other internal organs did not differ from those of control mice despite overexpression of GASP1. Unlike the myostatin-deficient animals or FS overexpressing mice, which exhibit both muscle hypertrophy and hyperplasia, we only observed an increase in myofiber size without a corresponding increase in myofiber number in surGasp1 animals. As the number of myofibers in muscle is largely determined during prenatal development, the overexpression of *Gasp1* does not seem to provide prenatal effect. However, we cannot preclude that the lack of hyperplasia is not the result of a too moderate expression of the transgene during embryonic and fetal stages, although *Gasp1* is strongly expressed in postnatal 3 days mice (Additional file 2). In the litterature, heterozygous mutations in the myostatin gene or surexpression of its propeptide have been reported to result also in hypertrophy and no hyperplasia [27]. Taken together, these results may be reflective of an incomplete inhibition of the myostatin. Moreover, Zhu *et al.*[28] have described that mice carrying a dominant negative form of myostatin preventing the release of the mature myostatin from the propeptide exhibited a significant increase in muscle mass that resulted from myofiber hypertrophy and not from myofiber hyperplasia. As Hill *et al*. [17] suggested, GASP1 may inhibit the propeptide proteolysis to keep the myostatin in a latent and inactive form. Such possible mechanism may explain the observed phenotype in the surGasp1 mice.

**Figure 8:**
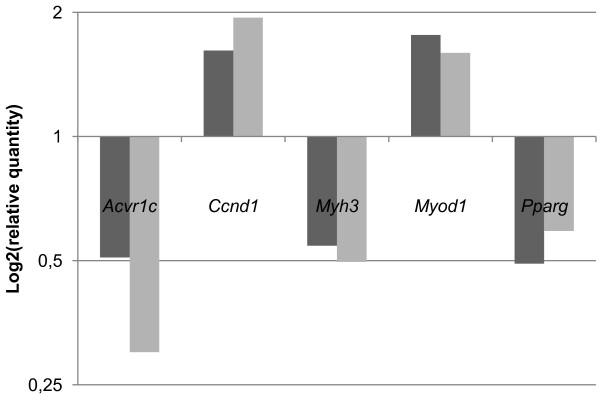
**Relative expression levels of genes involved in muscle development in quadriceps surGasp1 mice.** Using the TLDA technology, mRNA expression levels were determined by qRT–PCR relative to the reference genes *Gapdh*, *TFIID*, *Dffa* and *fcgrt* in quadriceps from surGasp1-20 (dark grey, n = 4) and surGasp1-06 (light grey, n = 4) animals. Calibration value: wild-type sample = 1.

The body weight increment in surGasp1 mice is visible at 3 weeks of age. However, during animal growth, the body weight difference between wild-type and transgenic mice does not increase, indicating that the effect of *Gasp1* overexpression may arise during the first 21 days of life, probably by satellite cells activity or AKT/FOXO dependent signalling pathway . To gain insights into the molecular mechanism, we performed an expression array analysis of 91 genes involved in muscle development. From those, only 5 genes *Ccnd1* (cyclin D1), *Actvr1c* (*Alk7*), *Myh3*, *Myod1* and *Pparγ* were slightly down- or up-regulated in the surGasp1 mice (Figure 8, Additional file 3: Table 1). Cyclin D1, an important component of the cell cycle machinery has been shown to be a major intracellular target for myostatin during myostatin-induced G1 cell cycle arrest and proliferation inhibition [29]. Ji et *al.*[30] showed that myostatin blocked the recruitment of p300 to the cyclin D1 promoter, resulting in the silencing of cyclin D1 gene expression. The increase of *Ccnd1* expression level in the surGasp1 mice is in accordance with those previous studies. The analysis of the *Gasp1* promoter revealed the presence of four PPARγ binding sites. A negative feedback loop may exist and could explain the down-regulation of *Ppar*γ in the mutant mice. Finally, most of the observed variations are related to an activation of the canonical Wnt/β-catenin signalling pathway and are consistent with observations in GDF8-null mice [31]. The mRNA expression levels of *Pax3* and *Pax7* genes which are expressed by postnatal satellite cells [32] or members of AKT/FOXO pathway remained unchanged in surGasp1 mice when compared to wildtype. However, the activation or repression of this pathway is essentially due to postranslational modification. Further studies are needed to understand the relevance of the AKT/FOXO signalling in the surGasp1 phenotype.

In mammals, myofibers are mainly classified into glycolytic and oxidative fibers based on their metabolic profiles. In mice, fast glycolytic fibers express the type IIB MHC isoform whereas oxidative fibers express type I (slow fibers), the fibers expressing MHC IIA are capable of both oxidative and glycolytic metabolism. We could show that *Gasp1* overexpression does not significantly change fiber type distribution while a lack of myostatin results in an alteration in the fiber type composition [33-36]. Glycolytic (phosphofructokinase, lactate dehydrogenase) or oxidative (citrate synthase, isocitrate dehydrogenase, cytochrome c-oxidase) enzyme activities did not show any significant differences between wild-type and surgasp1-20 mice, confirming the above mentioned result (data not shown).

Interestingly, we do not detect a change in fat pad mass in our *Gasp1* transgenic mice. This result differs from the results reported for the myostatin knockout mice or transgenic mice overexpressing the myostatin propeptide in which a reduction in adiposity is observed [14,37,38]. Recent literature showed that myostatin inhibition in skeletal muscle, but not in adipose tissues, is primarily responsible for a decrease of fat mass [39]. This effect on fat pad could reflect a regulation of myostatin independant from GASP1.

## Conclusions

Taken together, our data provide definitive evidence of the role of *Gasp1 in vivo,* in particular in muscle. In our transgenic mice, estimation of the amount of GDF8 associating with GASP1 will be an important issue, thus providing mechanistic insights into this association. We are currently crossing our transgenic mice overexpressing *Gasp1* on the myostatin null background to investigate if GASP1 stimulates muscle growth by additional mechanisms independent of myostatin inhibition like it has been shown in transgenic mice overexpressing follistatin presenting a quadrupling of muscle mass [15]. The related protein, GASP2 has also been shown to be capable of binding myostatin although GASP2 was not detected as one of the proteins bound to endogenous myostatin in serum [17]. GASP2 appears to be similar to GASP1 in its ability to bind GDF8 or GDF11 *in vitro* but effects of GASP2 on other TGFβ ligands are not yet known [18]. Further research will be required to determine the exact roles of GASP2 play *in vivo* and its interactions with GASP1.

## Methods

### Animals

All mice were bred and housed in the animal facility of Limoges University under controlled specific pathogen free conditions (21°C, 12-h light/12-h dark cycle) with free access to standard mouse chow and tap water. All of the experimental procedures were carried out in accordance with the local ethics commission.

### Transgene construction and generation of transgenic lines

First strand cDNA was synthesized from 1μg of total muscle RNA using the SuperScript® III Reverse Transcriptase (Invitrogen) with oligodT primers. The coding sequence of murine *Gasp-1* was PCR-amplified from muscle cDNA using primers 5^′^-ATGTGTGCCCCAGGGTATCATCG-3^′^ located at the position 159-181 bp and 5^′^-TCATTGCAAGCCCAGGAAGTCCTT-3^′^ located at the position 1851-1874 bp (transcript sequence ENSEMBL ENSMUST00000061469). The 1716 bp fragment (GenBank accession number: JQ080910) was introduced into the expression vector pcDNA^TM^3.1/V5-His® TOPO (Invitrogen) to generate the psurGasp1 vector. Consequently, *Gasp1* is under the human cytomegalovirus immediate-early promoter/enhancer leading to a strong constitutive expression. In our construct, the GASP1 protein is not expressed as a fusion to the V5 epitope and polyhistidine tag since a stop codon is present at the 3^′^ end of *Gasp1* cDNA. A purified 3578 bp Sal1-NsiI fragment was microinjected into the male pronucleus of one-cell fertilized FVB/N embryos. Transgenic founders, or surGasp1 animals were identified by PCR of tail-extracted genomic DNA using primers fwdU2: 5^′^-AAGTACGCCCCCTATTGACG-3^′^ and revR1: 5^′^-CCAGAGGTTGGGGTTCATGT-3^′^. Founders bearing the transgene were bred to wild-type FVB/N mice to generate F1 offspring. Transgenic F1 mice were bred with other transgenic or wildtype FVB/N mice as necessary to maintain and expand the colony.

### Phenotyping of mice

Muscle weights were measured following dissection of 12-week-old mice. Individual muscles from both sides of the animal were taken and the average weight was used for each muscle. Body composition analysis to determine fat and muscle contents was performed on conscious mice at 10 weeks of age, using the EchoMRI-500™ whole body composition analyzer (Echo Medical Systems).

### Histological and morphometric analysis

Cryosections (14 μm) of *rectus femoris* muscles were prepared from frozen muscles of wildtype or surGasp1 12-week-old mice. Transverse sections were processed for hematoxylin and eosin staining. For cross sectional area measurement and fiber counting, muscles were stained with a reticulin silver staining kit (04-040801, Bio-Optica Milano S.p.A). Fiber typing was performed using the myosin ATPase method (pH 4.6) allowing differentiating 3 fiber types: I, IIA and IIB. For morphometric analysis, the muscle fiber sizes were measured with Image J software.

### Quantitative PCR

Total RNA was isolated from spleen, kidney, liver, heart, lung, brain and skeletal muscles dissected from mice of various ages (8 to 12 weeks, see figures legend) using TRIzol (Invitrogen) according to the manufacturer’s instruction and relative RNA concentration was determined by spectrophotometric analysis. 1.5 μg of total RNA was reverse-transcribed into DNA using the high-capacity cDNA reverse transcription kit (Applied Biosystems). Real-time PCR was performed in triplicate using 50 ng of cDNA for each sample. Relative amounts of transcripts were determined using Taqman probes specific for *Gasp1* (Mm00725281_m1), *Gapdh (*Mm99999915_g1), *TfIID* (Mm00446973_m1) on an ABI PRISM^©^ 7900 system (Applied Biosystems). Relative mRNA expression values were calculated by the ΔΔCt method with normalization of each sample to the average change in cycle threshold value of the controls. TLDA (Taqman low density array, Applied Biosystems) assays were performed based on the same above conditions, except 100 ng cDNA was used for each fill reservoir. The selected target genes analysed are listed in the Additional file 3: Table 1.

### Genotyping assay

SurGasp1 F1 animals were genotyped by checking the presence of the CMV sequence using specific primers (fwdU2: AAGTACGCCCCCTATTGACG and revR1: CCAGAGGTTGGGGTTCATGT). These F1 animals are used as calibrator in the next step of the qPCR assay, i.e. their CMV or *Gasp1* copy number is arbitrarily defined as 1. Then, SYBR Green–based real-time PCR was carried out by 3 amplifications for each sample (F2 mice) using the ABI PRISM® 7900 system (Applied Biosystems). One reaction that uses Gasp1-Fwd (CAGTCTCAATGGCACAGCTT) and Gasp1-Rev (GAGATTGTGGTGGCAGTGAC) primers was designed to yield a 148 bp product that corresponds to Gasp1 exon 2, the second reaction, by using CMV-Fwd (CCCACTTGGCAGTACATCAA) and CMV-Rev (GCCAAGTAGGAAAGTCCCAT) primers, was designed to yield a 123 bp product that corresponds to the CMV promoter. The last reaction by using CCR5-Fwd (GCACAAAGAGACTTGAGGCA) and CCR5-Rev (GTCATCTCTAGGCCACAGCA) was designed to yield a 81 bp product that corresponds to the CCR5 allele defining our reference gene. All the tests were done in triplicate. Each reaction was carried out in 20 μl of reaction mixture that contained SYBR^®^ Green master mixture, each of the forward and reverse primers at a final concentration of 300 nM, and 5 ng of purified DNA sample. The thermal profile began with incubations at 50°C for 2 min and 95°C for 10 min followed by 40 cycles of amplification alternating between 95°C for 15 sec and 60°C for 1 min. The SYBR® Green fluorescent signal was obtained once per cycle at the end of the extension step. After amplification, melting curve analysis was performed by heating the PCR products to 95°C for 15 sec, then cooling it to 60°C for 15 sec, followed by a linear temperature increase to 95°C at a rate of 0.3°C/sec while continuously monitoring the fluorescent signal. Data were analyzed by the standard software, SDS 2.3, and RQ manager 1.2, included with the real-time PCR system.

### Western blot analysis

Total proteins were extracted from spleen, kidney, liver, heart, pancreas, brain and some skeletal muscles dissected from 12-week-old mice using RIPA buffer (50 mM Tris, pH 8, 150 mM NaCl, 0.1% SDS, 1% NP-40, 0.5% sodium deoxycholate, and protease inhibitors). The Bradford assay was used to quantify protein concentrations at A_595nm_. The proteins extracted from tissues (50 μg) were mixed with Laemmli loading buffer and heated for 5 min at 95°C. The proteins were separated under denaturating conditions into a 10% polyacrylamide gel and then transferred to a Hybond C-Extra Nitrocellulose membrane (Amersham Biosciences). Unspecific binding was prevented using 5% non fat dry milk (w/v) in TBS-T0.1% buffer (50 mM Tris, 150 mM NaCl, pH 7.4, 0.1% Tween-20) for 1 h at room temperature. First antibodies were diluted in 2% non fat dry milk in TBS-T0.1% buffer [goat anti-GASP1 (AF2070, R&D Systems, 0.2 μg.ml^-1^); rabbit anti-GASP1 (HPA010953, Sigma, 0.3 μg.ml^-1^); rabbit anti-GASP1 directed against the peptide DCGEEQTRWFDAQANN (0.9 μg.ml^-1^); goat anti-GAPDH antibody (R&D Systems, 0.5 μg.ml^-1^)]. The diluted antibodies were incubated with membrane overnight at 4°C with constant agitation, followed by several washing steps in TBS-T0.1%. Blots were incubated with the secondary antibodies, horseradish peroxidase-coupled anti-goat IgG or anti-rabbit IgG (Dako) at a dilution of 1:2000 in 2% non fat dry milk in TBS-T0.1% buffer for 1 h at RT. After several washing steps in TBS-T0.1%, the immunoblots were processed by chemiluminescence detection (BM Chemiluminescence Western Blotting Substrate (POD), Roche Applied Science) and exposed to a film (Amersham Hyperfilm ECL, Amersham Biosciences).

### Statistical analysis

Unless indicated otherwise, data are presented as mean ± SEM. Data are considered significant with p < 0.05 by two-tailed Student’s t test analysis.

## Competing interests

The authors declare that there are no competing interests.

## Authors’ contributions

OM and CB: performed the experiments and analysed the data. KH: made the transgene construct. BP: carried out the microinjection experiments. MM: helped with histological analyses. LM: contributed in the establishment and the maintenance of the surGasp1 lines. JLV: provided comments and revisions to the manuscript. VB: directed the work, discussed results and wrote the manuscript. All authors have read and approved the manuscript.

## Supplementary Material

Additional file 1 **Fiber type distribution in quadriceps of surGasp-1 mice.** No significant variation in proportion of type I (dark grey) or type II fibers (light grey) was observed in surGasp1-20, surGasp1-06 mice when compared to wild-type. Data are expressed +/-SEM. Click here for file

Additional file 2 **Neonatal expression of *Gasp-1 *and myogenic regulatory factors.** mRNA expression levels were determined by qRT–PCR relative to the reference genes *Gapdh* and *TfIID* in extracts from head (dark grey, n = 3) or hindlimb (light grey, n = 3) of postnatal 3 days surGasp1-20 animals. The horizontal dashed line represents a twofold increase in expression level.* : p < 0.05 ; ** : p < 0.01. Click here for file

Additional file 3 **Table 1:** List of the 96 genes that were measured on the TaqMan Low Density Arrays. Click here for file
