# Supplementary figures and images for: Ubiquitous Gasp1 overexpression in mice leads mainly to a hypermuscular phenotype
Source: BMC Genomics. 2012 Oct 10;13:541. doi: 10.1186/1471-2164-13-541 (PMC3575399; doi:10.1186/1471-2164-13-541)

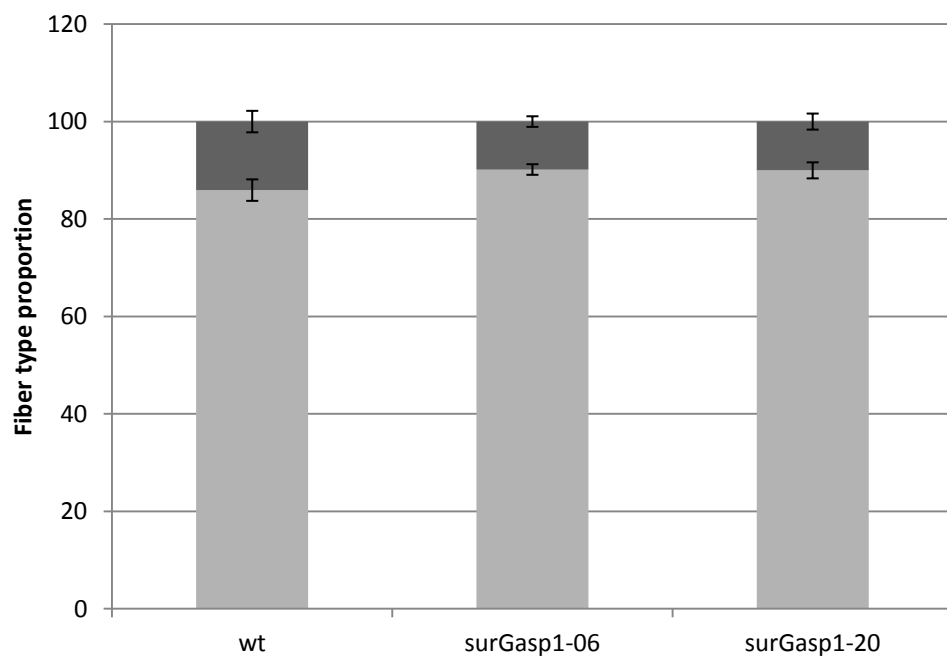

Supplement: Additional file 1 — Fiber type distribution in quadriceps of surGasp-1 mice. No significant variation in proportion of type I (dark grey) or type II fibers (light grey) was observed in surGasp1-20, surGasp1-06 mice when compared to wild-type. Data are expressed +/-SEM. [file 1471-2164-13-541-S1.pdf]

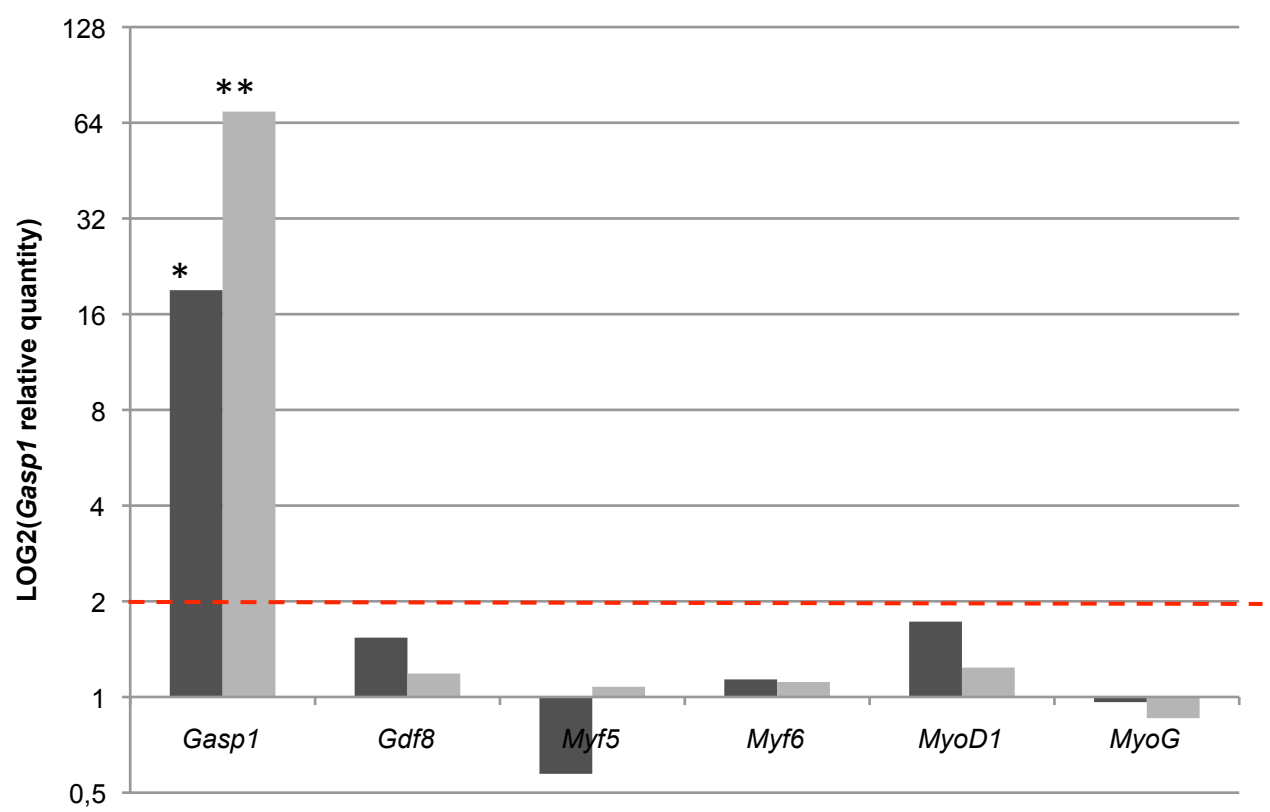

Supplement: Additional file 2 — Neonatal expression of Gasp-1 and myogenic regulatory factors. mRNA expression levels were determined by qRT–PCR relative to the reference genes Gapdh and TfIID in extracts from head (dark grey, n = 3) or hindlimb (light grey, n = 3) of postnatal 3 days surGasp1-20 animals. The horizontal dashed line represents a twofold increase in expression level.* : p < 0.05 ; ** : p < 0.01. [file 1471-2164-13-541-S2.pdf]
