# Supplementary material for: Ubiquitous Gasp1 overexpression in mice leads mainly to a hypermuscular phenotype
Source: BMC Genomics. 2012 Oct 10;13:541. doi: 10.1186/1471-2164-13-541 (PMC3575399; doi:10.1186/1471-2164-13-541)
Supplement: Additional file 3 — Table 1: List of the 96 genes that were measured on the TaqMan Low Density Arrays. [file 1471-2164-13-541-S3.pdf]

Table 1: List of the 96 genes that were measured on the TaqMan Low Density Arrays.

Assay	Type
18S-Hs99999901_s1	Control
2400001E08Rik-Mm01279964_g1	Target
Acvr1-Mm01331069_m1	Target
Acvr1b-Mm00475713_m1	Target
Acvr1c-Mm03023957_m1	Target
Acvr2a-Mm00431657_m1	Target
Acvr2b-Mm00431664_m1	Target
Aebp1-Mm00477402_m1	Target
Akt1-Mm01331626_m1	Target
Akt2-Mm02026778_g1	Target
Akt3-Mm00442194_m1	Target
Axin1-Mm01299063_m1	Target
Bmp2-Mm01340178_m1	Target
Bmp3-Mm00557790_m1	Target
Bmp4-Mm01321704_m1	Target
Casp9-Mm00516563_m1	Target
Ccnd1-Mm00432359_m1	Target
Ccne1-Mm00432367_m1	Target
Cdk2-Mm00443947_m1	Target
Cdk4-Mm00726334_s1	Target
Cdk6-Mm01311342_m1	Target
Ckm-Mm00432556_m1	Target
Ctnnb1-Mm00483033_m1	Target
Cyp2b10-Mm01972453_s1	Target
Dctn6-Mm00495994_m1	Target
Dffa-Mm00438410_m1	Control
Fbxo32-Mm00499523_m1	Target
Fcgrt-Mm00438887_m1	Control
Foxo1-Mm00490671_m1	Target
Foxo3-Mm01185722_m1	Target
Foxo4-Mm00840140_g1	Target
Fst-Mm00514982_m1	Target
Fstl3-Mm00473194_m1	Target
Gapdh-Mm99999915_g1	Control
Gdf11-Mm01159973_m1	Target
Gsk3b-Mm00444911_m1	Target
Igf1-Mm01228180_m1	Target
Igf1r-Mm00802831_m1	Target
Igf2-Mm00580426_m1	Target
Inha-Mm00439683_m1	Target
Inhbb-Mm01286587_m1	Target
Map2k2-Mm00445688_m1	Target
Map2k3-Mm00435950_m1	Target
Map2k4-Mm00436508_m1	Target
Map2k5-Mm00488652_m1	Target
Map2k6-Mm00803694_m1	Target
Mapk1-Mm00442479_m1	Target
Mapk10-Mm00436518_m1	Target
Mapk3-Mm01973540_g1	Target
Mapk7-Mm00839961_g1	Target

Mapk8-Mm00489514_m1	Target
Mapk9-Mm00444239_m1	Target
Mdm2-Mm01233136_m1	Target
Mstn-Mm01254559_m1	Target
Mtor-Mm00444968_m1	Target
Myf5-Mm00435125_m1	Target
Myf6-Mm00435126_m1	Target
Myh1-Mm01332489_m1	Target
Myh13-Mm01329439_m1	Target
Myh2-Mm01332564_m1	Target
Myh3-Mm01332463_m1	Target
Myh4-Mm01332518_m1	Target
Myh6-Mm00440359_m1	Target
Myh7-Mm00600555_m1	Target
Myh7b-Mm01249941_m1	Target
Myh8-Mm01329494_m1	Target
Myod1-Mm00440387_m1	Target
Myog-Mm00446194_m1	Target
Nodal-Mm00443040_m1	Target
Pak2-Mm01170646_m1	Target
Pax3-Mm00435493_m1	Target
Pax7-Mm00834079_m1	Target
Pdpk1-Mm00440707_m1	Target
Pparg-Mm01184322_m1	Target
Rps6kb1-Mm01310033_m1	Target
Smad1-Mm00484721_m1	Target
Smad2-Mm00487530_m1	Target
Smad3-Mm01170760_m1	Target
Smad4-Mm03023996_m1	Target
Smad5-Mm03024001_g1	Target
Smad6-Mm00484738_m1	Target
Smad7-Mm00484742_m1	Target
Smad9-Mm00649885_m1	Target
Smurf1-Mm00547102_m1	Target
Smurf2-Mm03024086_m1	Target
Syp-Mm00436850_m1	Target
Tbp-Mm00446973_m1	Control
Tgfb1-Mm01178819_m1	Target
Tgfb2-Mm00436955_m1	Target
Tgfbr1-Mm00436964_m1	Target
Tgfbr2-Mm00436977_m1	Target
Tgfbr3-Mm00803538_m1	Target
Tnf-Mm00443258_m1	Target
Trim63-Mm01185221_m1	Target
Wfikkn1-Mm01308311_m1	Target
Wfikkn2-Mm00725281_m1	Target
